# CNOT2 /c-Myc/STAT3 signaling is critically involved in glycolysis mediated apoptosis of benzyl isothiocyanate in hepatocellular carcinoma

**DOI:** 10.1038/s41598-026-38416-8

**Published:** 2026-02-02

**Authors:** Wonil Koh, Su-Yeon Park, Bonglee Kim, Bum-Sang Shim, Sung-Hoon Kim

**Affiliations:** Cancer Molecular Target Herbal Research Lab, College of Korean Medicine, Graduate School, Kyunghee University, 1 Hoegi-Dong, Dongdaemun-Gu, Seoul, 02447 Republic of Korea

**Keywords:** Hepatocellular carcinoma, Benzyl isothiocyanate, Apoptosis, Glycolysis, CNOT2, c-Myc, STAT3, Cancer, Cell biology, Molecular biology, Oncology

## Abstract

Although benzyl isothiocyanate (BITC), a major compound found in cruciferous vegetables, has been reported to exert antitumor effects in various cancers, its apoptotic mechanism remains unclear. This study aimed to elucidate the apoptotic mechanism of BITC by investigating its role in inhibiting Warburg effect in hepatocellular carcinoma (HCC) cells. BITC suppressed cell proliferation, increased the sub-G1 population, Annexin V/PI and reduced the expression of pro-poly (ADP-ribose) polymerase (pro-PARP), pro-caspase-3, CCR4-NOT transcription complex subunit 2 (CNOT2), c-Myc, signal transducer and activator of transcription 3 (STAT3), and phosphorylated Janus kinase 1 (p-JAK1) in SK-Hep1 and Huh7 HCC cell lines. Notably, knockdown of STAT3 or its upstream regulator CNOT2 further enhanced BITC-induced apoptosis, as evidenced by decreased pro-PARP and pro-caspase-3 expression in SK-Hep1 cells. Additionally, BITC attenuated the expression of hexokinase 2 (HK2), pyruvate kinase M2 (PKM2), and lactate dehydrogenase (LDH) along with reduced LDH production and glucose in SK-Hep1 and Huh7 cells. However, treatment of pyruvate or overexpression of CNOT2 or c-Myc reversed the capacity of BITC to reduce the expression of HK2, pro-caspase-3, and pro-PARP in SK-Hep1 cells. Immunoprecipitation assays further revealed that BITC disrupted the interactions between CNOT2 and STAT3 or c-Myc. Collectively, these findings suggest that the CNOT2/c-Myc/STAT3 signaling axis plays a critical role in glycolysis mediated apoptosis of BITC in HCC cells.

**Supplementary Information:**

The online version contains supplementary material available at 10.1038/s41598-026-38416-8.

## Introduction

Liver cancer ranks as the fourth leading cause of death and the sixth most prevfalent cancer worldwide^1^. Statistically hepatocellular carcinoma (HCC) accounts for approximately 90% of all primary liver cancers with over 1 million new cases diagnosed annually^2^. Although various treatments—including surgical resection, transcatheter arterial chemoembolization (TACE), and molecular targeted therapies—have been implemented in recent decades, the recurrence rate of HCC remains high.

CCR4-NOT Transcription Complex Subunit 2 (CNOT2) is known to bind c-Myc oncogene or midline1 interacting protein 1 (MID1IP1) in liver cancer progression and fatty liver^3^. Also, He et al. reported that neuropilin and tolloid-like 2 (NETO2) promote HCC progression via STAT3/c-Myc pathway, since c-Myc is a well-known oncogene^4^ and STAT3 is critically involved in tumor progression as a transcription factor and an downstream of Janus kinase (JAK)^5^.

Aerobic glycolysis, so called as Warburg effect or is known to enhance tumor progression and resist apoptosis by high mitochondrial reactive oxygen species (ROS) generation as a “Hallmark of Cancer^6,7^.” Recent evidence reveals that the PI3K/Akt signaling plays a critical role in promoting Warburg effect, by phosphorylation of metabolic enzymes, such as PFKB3/4, GLUT1, HK2, PKM2, and their molecular networks including glycogen synthase kinase 3 (GSK3), HIF-1α, mammalian target of rapamycin complex 1 (mTORC1), Myc and forkhead box O (FOXO)^8^.

Isothiocyanates including phenylethyl isothiocyanate, allyl isothiocyanate and BITC are known an anticancer compound derived from cruciferous plants such as broccoli, brussels sprouts, cabbage, kale, mustard, and rocket^9^. BITC as an aromatic isocyanate has been known to have anticancer effect in acute myeloid leukemia^10^, breast cancer stem cells^11^, oral squamous cancer cells^11^ and Gefitinib-resistant NCI-H460 human lung cancer cells^12^. Also, BITC showed anticancer property by inhibiting Akt/MAPK and activating Nrf2/ARE signaling pathways in HepG2 cells^13^. Similarly, BITC exerted antitumor effect in diethylnitrosamine treated tumor model via HGF/pAkt/STAT3 axis^14^ and also increased TIMP-2 expression and inhibited MMP-2 and MT1-MMP in SK-Hep1 cells^15^. Nonetheless, there is no report on the antitumor mechanism of BITC in association with Warburg effect so far. Hence in the current project, apoptotic and anti-Warburg mechanisms of BITC were explored in SK-Hep1 and Huh7 hepatocellular carcinoma cells.

## Results

### Cytotoxic effect of BITC in SK-Hep1 and Huh7 hepatocellular cancer cells

**Fig. 1:**
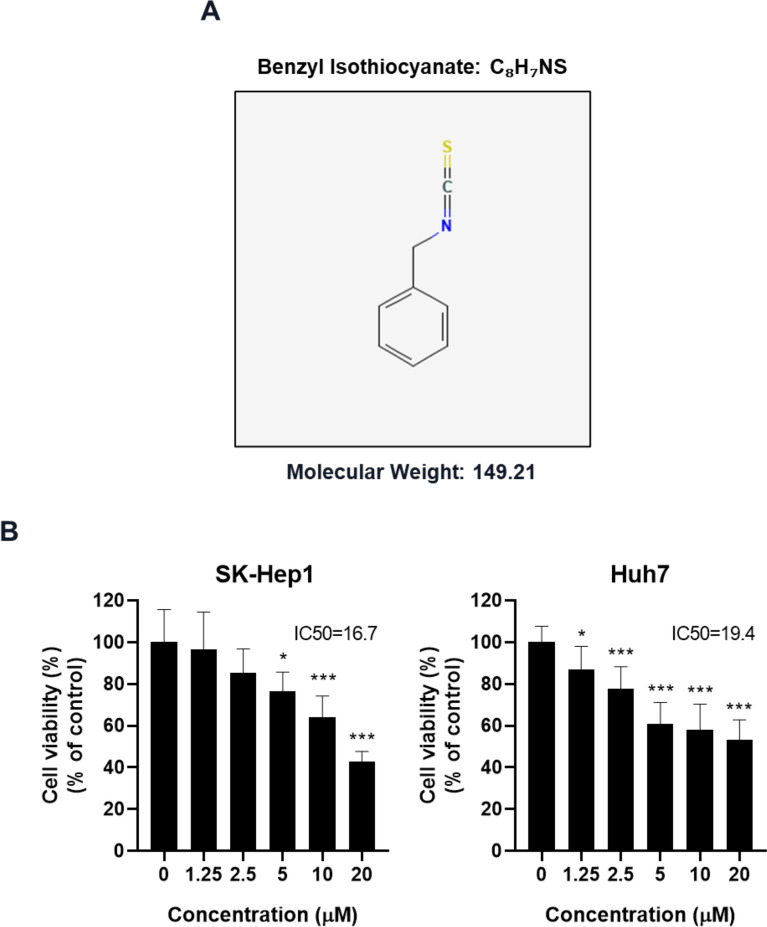
Effect of BITC on cytotoxicity in SK-Hep1 and Huh7 cells. (**A**) Chemical structure of BITC (Molecular weight = 149.21 g/mol) (**B**) SK-Hep1 and Huh7 cells were exposed to various concentrations of BITC for 24 h and cell viability was assessed by using MTT assay. Data represent means ± SD. *p < 0.05, ***p < 0.001 vs untreated control. All experiments were performed using biological triplicates and independently repeated three times**.**

To evaluate the cytotoxic effect of benzyl isothiocyanate (BITC) (Fig. 1A), an MTT assay was performed on SK-Hep1 and Huh7 hepatocellular carcinoma cells. Cells were treated with increasing concentrations of BITC (0, 1.25, 2.5, 10, and 20 μM) for 24 h. BITC significantly reduced cell viability in both SK-Hep1 and Huh7 cells in a concentration-dependent manner (Fig. 1B).

### BITC decreased the expression of pro-PARP and pro-caspase3 and increased subG1 population in SK-Hep1 and Huh7 cells

**Fig. 2:**
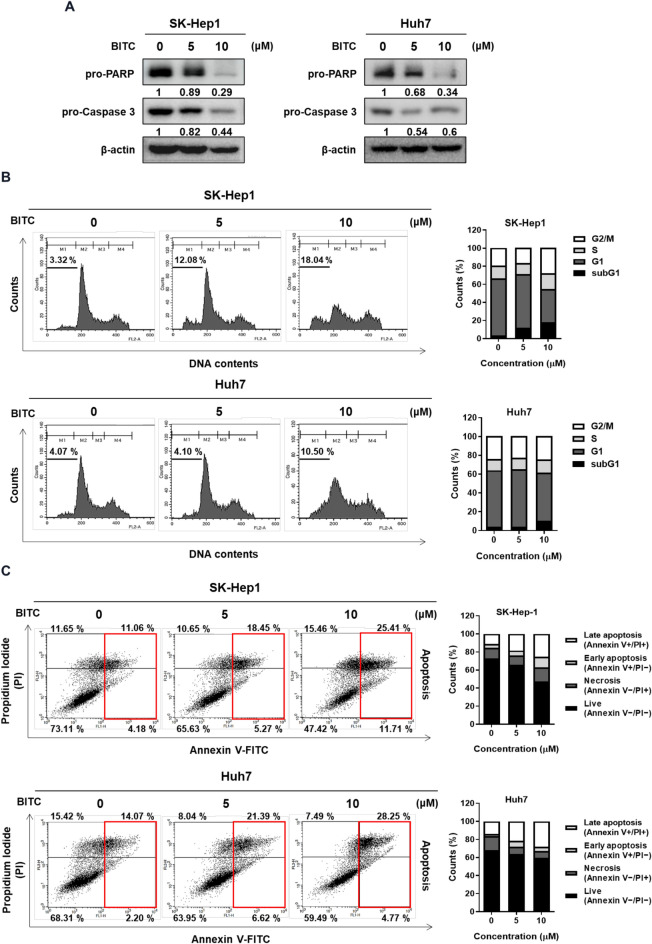
Effect of BITC on pro-PARP and pro-caspase and sub-G1 population in SK-Hep1 and Huh7 cells. (**A**) Effect of BITC on pro-PARP and pro-caspase in SK-Hep1 and Huh7 cells by Western blotting. Band intensities were quantified and normalized to β-actin. (**B**) Effect of BITC on sub G1 accumulation in SK-Hep1 and Huh7 cells by flow cytometry analysis. (**C**) Effect of BITC on apoptosis in SK-Hep1 and Huh7 cells by Annexin V/PI double staining assay. **p < 0.01, ***p < 0.001 vs untreated control. All experiments were performed using biological triplicates and independently repeated three times**.**

To investigate the apoptotic effects of BITC, Western blot analysis and cell cycle profiling were performed in BITC-treated SK-Hep1 and Huh7 cells. As shown in Fig. 2A, BITC treatment led to a reduction in the expression levels of pro-caspase-3 and pro-PARP compared to untreated controls. Additionally, BITC significantly increased the sub-G1 cell population and apoptotic portion such as early and late apoptosis for Annexin V/PI staining in both cell lines, indicating enhanced apoptosis (Fig. 2B, C). It is well known that early apoptosis is induced by positive Annexin V/negative PI staining and late apoptosis is induced by positive Annexin V/positive PI staining, while primary necrosis is induced by only PI staining^16^.

### Effect of BITC on CNOT2 and pSTAT3 and their binding in SK-Hep1 and Huh7 cells

**Fig. 3:**
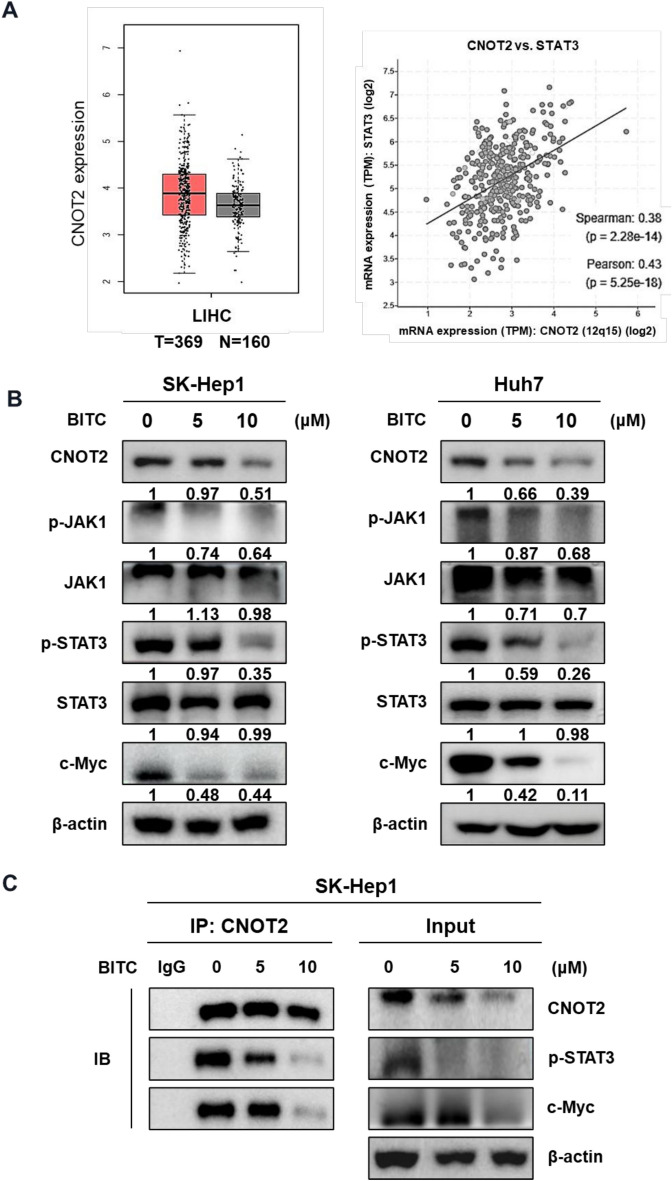
Effect of BITC on CNOT2 and pSTAT3 and their binding in SK-Hep1 and Huh7 cells. (**A**) Overexpression of CNOT2 in liver cancer patients implies poor survival rate along with original regression ratio with STAT3. (**B**) Effect of BITC on CNOT2 and pSTAT3 in SK-Hep1 and Huh7 cells. Band intensities were quantified and normalized to β-actin. (**C**) Effect of BITC on the binding between CNOT2 and STAT3 or c-Myc in SK-Hep1 cells. All experiments were performed using biological triplicates and independently repeated three times**.**

CNOT2 was found to be overexpressed at the mRNA level in liver cancer (LIHC) patients with poor survival outcomes compared to the normal control group (Fig. 3A). Although a weak ordinal regression correlation (r = 0.38) was observed between CNOT2 and STAT3 expression, BITC treatment led to a marked reduction in phosphorylated JAK1 (pJAK1), phosphorylated STAT3 (pSTAT3), and c-Myc levels in SK-Hep1 and Huh7 cells (Fig. 3B). Furthermore, immunoprecipitation assays demonstrated that BITC disrupted the interactions between CNOT2 and STAT3, as well as between CNOT2 and c-Myc, in SK-Hep1 cells (Fig. 3C).

### Depletion of STAT3 or CNOT2 enhances the apoptotic capacity of BITC in SK-Hep1 cells

**Fig. 4:**
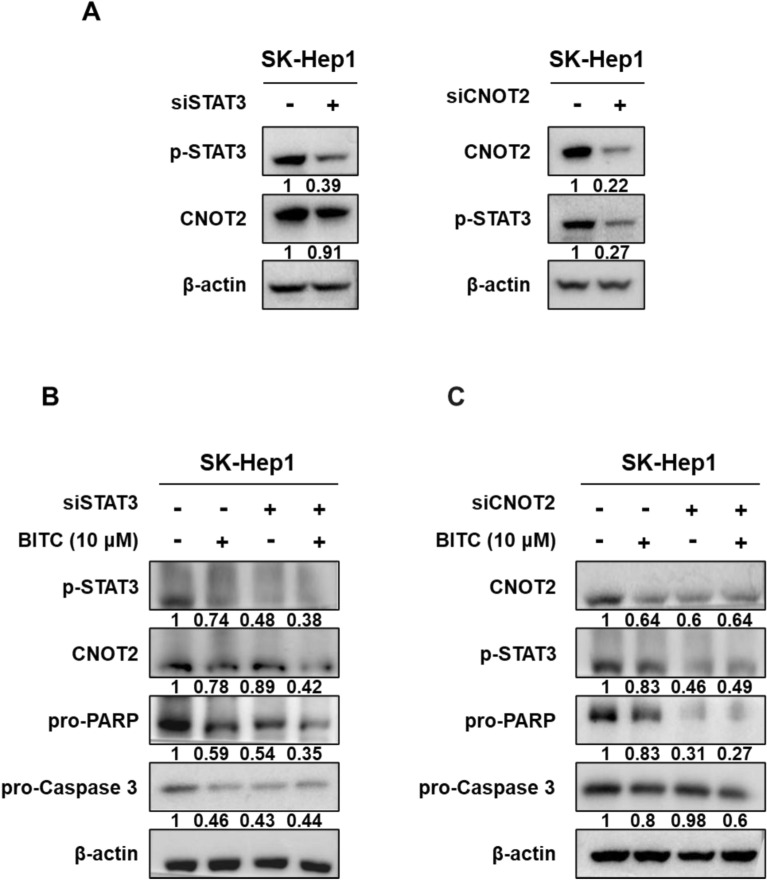
Depletion of STAT3 or CNOT2 enhances apoptotic effect of BITC in SK-Hep1 cells. (**A**) Effect of STAT3 or CNOT2 depletion in BITC treated SK-Hep1 cells. (**B**) Effect of STAT3 depletion on pro-PARP and pro-caspase3 in BITC treated SK-Hep1 cells. (**C**) Effect of CNOT2 depletion on pro-PARP and pro-caspase3 in BITC treated SK-Hep1 cells. Band intensities were quantified and normalized to β-actin. All experiments were performed using biological triplicates and independently repeated three times**.**

To confirm the regulatory roles of STAT3 and CNOT2, RNA interference was performed in SK-Hep1 cells. Knockdown of STAT3 did not alter CNOT2 expression, whereas knockdown of CNOT2 led to decreased STAT3 expression, suggesting that CNOT2 functions upstream of STAT3. Furthermore, silencing of either STAT3 or CNOT2 enhanced the reduction of pro-caspase-3 and pro-PARP expression, indicating their involvement in apoptosis regulation (Fig. 4A, B).

### BITC inhibits Warburg effect in SK-Hep1 and Huh7 cells, which was reversed by pyruvate treatment or overexpression of CNOT2 or c-Myc

**Fig. 5:**
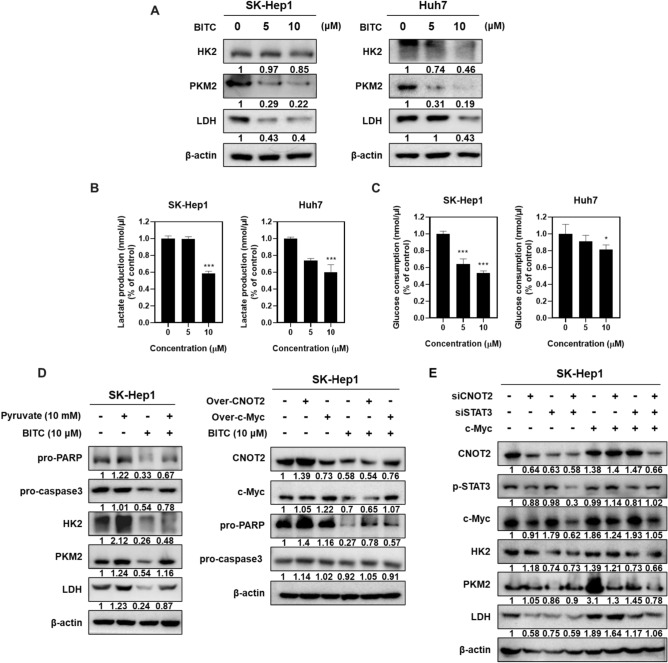
Effect of BITC on Warburg effect in SK-Hep1 and Huh7 cells. (**A**) Effect of BITC on Warburg effect proteins in SK-Hep1 and Huh7 cells. (**B**) Effect of BITC on LDH production in SK-Hep1 and Huh7 cells. STAT3 or CNOT2 depletion vs untreated control. ***p < 0.001 vs untreated control. (**C**) Effect of BITC on glucose consumption in SK-Hep1 and Huh7 cells. *p < 0.05, ***p < 0.001 vs untreated control. (**D**) Effect of pyruvate treatment or overexpression of CNOT2 or c-Myc on apoptosis and glycolysis proteins in BITC treated SK-Hep1 cells. (E) Effect of siSTAT3 or/and siCNOT2 on HK2, PKM2 and LDH in SK-Hep1 cells transfected with or without c-Myc overexpression plasmid. Cells were co-transfected with control vector, siSTAT3, siCNOT2 and siCNOT2 + c-Myc OE (overexpression) plasmids as indicated in each lane. Band intensities were quantified and normalized to β-actin. All experiments were performed using biological triplicates and independently repeated three times**.**

To investigate the role of Warburg effect in BITC-induced apoptosis in HCC cells, Western blot analysis was conducted in SK-Hep1 and Huh7 cells. BITC significantly reduced the expression of key glycolytic markers, including HK2, PKM2, and LDH (Fig. 5A). Consistently, BITC also decreased LDH production and glucose consumption in both cell lines compared to the untreated control (Fig. 5B, C). Conversely, supplementation with pyruvate impaired the ability of BITC to suppress the expression of pro-PARP, pro-caspase-3, HK2, PKM2, and LDH in SK-Hep1 cells. Moreover, overexpression of CNOT2 or c-Myc reversed the BITC-induced downregulation of pro-caspase-3 and pro-PARP in SK-Hep1 cells (Fig. 5D). Likewise, depletion of CNOT2 and/or Stat3 reversed the effect of c-Myc overexpression to enhance glucose related proteins such as PKM2, and LDH in SK-Hep1 cells (Fig. 5E) Exogenous pyruvate has been used as a functional metabolic rescue agent to clarify whether BITC-induced apoptosis is linked to inhibition of glycolytic flux. Pyruvate can bypass upstream glycolytic blockade by directly fueling mitochondrial metabolism, thereby partially restoring ATP production^17^. In addition, pyruvate was known to have antioxidant properties to mitigate oxidative stress–induced apoptosis^18,19^. Therefore, the ability of pyruvate to reverse BITC-induced effects likely reflects a combination of metabolic energy compensation and redox homeostasis rather than activation of an independent signaling pathway. These findings support the conclusion that BITC-mediated apoptosis is mechanistically coupled to metabolic reprogramming and disruption of the Warburg effect.

## Discussion

Phenethyl isothiocyanate, allyl isothiocyanate, benzyl isothiocyanate and sulforaphane are isothiocyanate derivatives abundant in cruciferous vegetables^20^. Although the antitumor effect of BITC have been reported in breast, bladder, lung, colon cancers^21–24^, its impact on glycolysis has not been previously explored. In the present study, we investigated the apoptotic mechanism of BITC in relation to aerobic glycolysis in hepatocellular carcinoma (HCC) cells. BITC inhibited cell proliferation, increased the sub-G1 population (indicative of apoptosis), and reduced the expression of pro-caspase-3 and pro-PARP in SK-Hep1 and Huh7 cells, supporting its apoptotic potential. Consistently, Zakaria et al.^14^ demonstrated the antitumor effects of BITC in HCC models via modulation of the HGF/pAkt/STAT3 axis and VEGF signaling. Additionally, Nakamura et al.^25^ showed that BITC induces apoptosis in rat liver epithelial RL34 cells through the mitochondrial death pathway.

CNOT2 has been well characterized as an oncogene^26^ and a known binding partner of c-Myc^3^ in several cancers. Similarly, activation of the JAK/STAT3 signaling pathway plays a pivotal role in inflammation, immune regulation, and tumor progression^27^. In our study, BITC markedly reduced the expression of CNOT2, c-Myc, phosphorylated STAT3 (p-STAT3), and phosphorylated JAK1 (p-JAK1) in both SK-Hep1 and Huh7 cells. Moreover, knockdown of STAT3 or its upstream regulator CNOT2 enhanced the apoptotic effects of BITC, further suppressing the expression of pro-caspase-3 and pro-PARP. These findings highlight the critical role of CNOT2 and STAT3 in BITC-induced apoptosis. Supporting this mechanism, BITC has also been shown to exert antitumor activity via STAT3 inhibition in pancreatic^28^ and breast cancer^29^.

Emerging evidence suggests that aerobic glycolysis, or the Warburg effect, contributes to cancer progression by upregulating key glycolytic regulators such as HK2, PKM2, and LDH^30–32^. Shi et al. reported that high glucose levels promote cancer progression, including metastasis and chemoresistance^33^. Furthermore, Ciscato et al. demonstrated that mitochondrial localization of HK2 in mitochondria-associated membranes (MAMs) facilitates neoplastic progression. Consistent with these findings, our data show that BITC suppressed the expression of HK2, PKM2, and LDH, and also reduced LDH production in SK-Hep1 and Huh7 cells, suggesting an anti-Warburg effect of BITC.

Regarding the relationship between glycolytic suppression and apoptosis, our findings reveal that BITC-induced disruption of the CNOT2/c-Myc/STAT3 axis leads to early suppression of glycolytic enzymes, resulting in metabolic stress, ATP depletion, and mitochondrial dysfunction, which subsequently trigger apoptotic signaling. Alternatively, glycolytic inhibition may occur as a downstream consequence of apoptosis, since caspase activation and global translational repression during apoptosis can reduce metabolic enzyme expression. A third possibility involves a feed-forward loop, in which early metabolic impairment initiates apoptosis that further amplifies glycolytic shutdown during late apoptotic or secondary necrotic stages.

Supporting a contributory role of metabolic regulation upstream of apoptosis, we observed that treatment with pyruvate—the end product of glycolysis—or overexpression of CNOT2 or c-Myc impaired the ability of BITC to downregulate HK2, pro-caspase-3, and pro-PARP, strongly implicating the involvement of the CNOT2/c-Myc axis in BITC-mediated metabolic reprogramming. Furthermore, immunoprecipitation assays revealed that BITC disrupted the interactions between CNOT2 and STAT3 or c-Myc, suggesting that BITC directly interferes with these oncogenic signaling complexes.

**Fig. 6:**
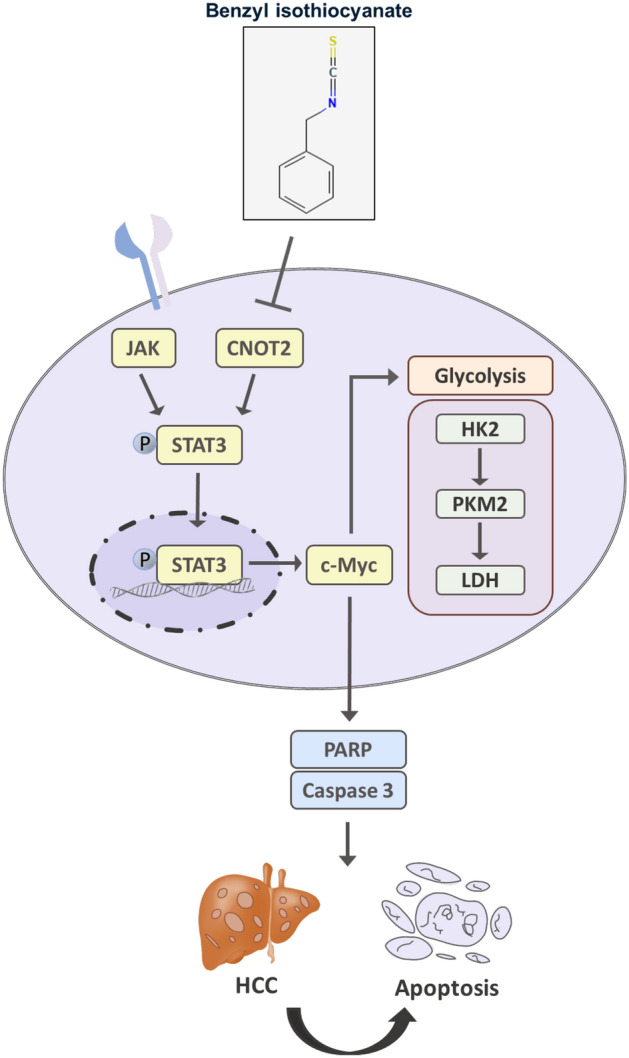
Scheme on mechanism of BITC via CNOT2/c-Myc/STAT3 signaling in HCCs.

Collectively, these findings demonstrate that BITC exerts both pro-apoptotic and anti-Warburg effects in HCC cells through modulation of the CNOT2/c-Myc/STAT3 signaling axis (Fig. 6), highlighting its potential as a promising anticancer agent for hepatocellular carcinoma. Nevertheless, this study is limited by the use of only two HCC cell lines and the lack of in vivo validation. Previous studies have demonstrated the antitumor efficacy of BITC in animal models, including pancreatic cancer xenografts^34^ and MDA-MB-231 breast cancer xenografts^35^. In addition, Cho et al.^36^ reported that dietary BITC reduced the expression of Ki-67, cyclin A, cyclin D1, and CDK2 in prostatic tissues, while Pore et al.^37^ showed that oral administration of BITC (10 mg/kg) inhibited MDA-MB-231-induced skeletal metastasis by approximately 81%. Thus, future studies are warranted to validate the CNOT2/c-Myc/STAT3-mediated glycolytic regulation of BITC in additional HCC models and in vivo systems, including subcutaneous or orthotopic xenografts and chemically induced HCC models, as well as to evaluate pharmacodynamic properties and potential toxicity to further support its translational relevance.

## Methods

### Cell culture

Human hepatocellular carcinoma cell lines, specifically Sk-Hep1 and Huh7, were obtained from the American Type Culture Collection (ATCC, Manassas, VA, USA). The Sk-Hep1 cells were maintained in Dubbeco Modified Eagle’s Medium (DMEM, Cat. No. LM 001–05, WelGENE, Republic of Korea), whereas Huh7 cells were cultured in RPMI-1640 medium (Cat. No. LM 011–01, WelGENE, Republic of Korea). Cultures were kept at 37 °C in a humidified atmosphere containing 5% CO₂, with the media supplemented with 10% heat-inactivated fetal bovine serum (FBS) and antibiotics (100 U/mL penicillin and 100 μg/mL streptomycin).

### Assessment of cell viability

The cytotoxic effects of BITC were evaluated using the MTT assay. Cells (10,000 cells per well) of Sk-Hep1 and Huh7 were seeded into 96-well plates and exposed to varying concentrations of BITC for 24 h. Following treatment, cells were incubated with 1 mg/mL MTT reagent (Sigma-Aldrich) for 2 h. The formazan product was dissolved overnight with MTT lysis buffer, and absorbance was measured at 570 nm using a microplate reader (Molecular Devices, USA). Cell viability percentages were calculated relative to untreated control samples.

### Transfection of siRNA and plasmid constructs

For gene knockdown and overexpression studies, HCC cells were plated overnight and transfected with siRNAs targeting CNOT2 or STAT3, or with plasmids overexpressing CNOT2 or c-Myc, all purchased from Bioneer (Korea). Transfections were performed using INTERFERin® reagent (Polyplus, France) at a final concentration of 40 nM, following the manufacturer’s instructions. Cells were incubated for 48 h post-transfection before subsequent analyses. Plasmids for overexpression were also obtained from Addgene (MA, USA) and transfected using Turbofect reagent (Thermo Fisher Scientific, USA).

### Cell cycle distribution analysis

Cells (1 × 10^5^ per milliliter) treated with BITC for 24 h were washed with cold PBS, fixed in 75% ethanol at -20 °C, and stored overnight. Fixed cells were then treated with RNase A (10 mg/mL) at 37 °C for 1 h, followed by staining with propidium iodide (50 μg/mL) for 30 min in the dark. DNA content was analyzed via flow cytometry using a FACSCalibur instrument (BD Biosciences), and data were processed with CellQuest Pro software v5.2 (BD Biosciences; https://www.bdbiosciences.com).

### Annexin V / PI staining assay

Cells (1 × 10⁶ cells/mL) from Huh7 and SK-Hep-1 cell lines were treated with various concentrations of BITC for 24 h. Then apoptotic cells were quantified by double staining with Annexin V–FITC and propidium iodide (PI) according to the manufacturer’s instructions, followed by flow cytometric analysis to distinguish viable, early apoptotic, late apoptotic, and necrotic populations.

### Western blot analysis

Cells (1 × 10⁶ per mL) from Huh7 and Sk-Hep1 lines were treated with different concentrations of BITC for 24 h, lysed in buffer containing 50 mM Tris–HCl (pH 7.4), 150 mM NaCl, 1% Triton X-100, 0.1% SDS, along with protease and phosphatase inhibitors. Lysates were clarified by centrifugation at 14,000 × g for 20 min at 4 °C. Protein concentrations were measured with a BCA assay (Bio-Rad). Equal amounts of protein were resolved on 4–12% NuPAGE Bis–Tris gels (Thermo Fisher Scientific) and transferred onto PVDF membranes. Blots were probed with primary antibodies against CNOT2 (#34,214, 1:1000; Cell Signaling Technology, MA, USA), c-Myc (ab32072, 1:1000, Abcam, Cambridge, UK), JAK1 (#3332, 1:1000; Cell Signaling Technology, MA, USA), STAT3 (#12,640, 1:1000; Cell Signaling Technology, MA, USA), phospho-JAK1 (#3331, 1:1000; Cell Signaling Technology, MA, USA), phospho-STAT3 (#4441, 1:1000; Cell Signaling Technology, MA, USA), pro-PARP (#9542, 1:1000; Cell Signaling Technology, MA, USA), pro-caspase-3 (#9662, 1:1000; Cell Signaling Technology, MA, USA), HK2 (#2106, 1:1000; Cell Signaling Technology, MA, USA) , PKM2 (#4053, 1:1000; Cell Signaling Technology, MA, USA), LDH (SC-133123, 1:1000; Santa Cruz, CA, USA) and β-actin (A1978, 1:10,000; Sigma-Aldrich, MO, USA). Secondary HRP-conjugated antibodies were applied, and bands visualized with an ECL detection kit.

### Metabolic measurements

Following BITC treatment for 24 h, culture media from Sk-Hep1 and Huh7 cells were collected. Lactate levels were quantified using ELISA kits (K-607, BioVision, CA, USA), according to the manufacturer’s instructions.

### Co-immunoprecipitation assay

SK-Hep1 cells treated with BITC were lysed in a buffer composed of 50 mM Tris–HCl (pH 7.4), 0.1% SDS, 150 mM NaCl, 1% Triton X-100, along with 1 mM each of NaF, EDTA, Na3VO4, and a protease inhibitor cocktail. The lysates were incubated with antibodies specific to CNOT2 to facilitate immunoprecipitation. Protein G/A agarose beads (Santa Cruz Biotechnology) were then added to capture the immune complexes. After washing and elution, the precipitated proteins were analyzed by immunoblotting using appropriate primary antibodies to detect interacting proteins.

### Data analysis and statistics

All experimental data were processed using GraphPad Prism 8.0 software (GraphPad Software, CA, USA; https://www.graphpad.com). Results are presented as mean ± standard deviation (SD). Differences between two groups were assessed by Student’s t-test, with a p-value of less than 0.05 considered statistically significant.

## Supplementary Information

Supplementary Information.

## Data Availability

All data generated or analyzed during this study are included in this manuscript or supplementary information files. Additional raw data, such as flow cytometry files and quantitative datasets are available from the corresponding author upon request. Further enquiries can be directed to the corresponding author (sungkim7@khu.ac.kr).

## Abbreviations

BITC: Benzyl isothiocyanate
BSA: Bovine serum albumin
Caspase: Cysteine aspartyl-specific protease
CNOT2: CCR4-NOT transcription complex subunit 2
ECL: Enhanced chemiluminescence
FACS: Fluorescence-activated cell sorting
FBS: Fetal bovine serum
HK2: Hexokinase 2
LDH: Lactate dehydrogenase
MTT: 3-(4,5-Dimethylthiazol-2yl)-2,5-diphenyltetrazolium bromide
OD: Optical density
PARP: Poly (ADP-ribose) polymerase
PBS: Phosphate buffered saline
PKM2: Pyruvate kinase M2
SD: Standard deviation
SDS: Sodium dodecylsulfate
STAT3: Signal transducer and activator of transcription 3
