# Supplementary material for: CNOT2 /c-Myc/STAT3 signaling is critically involved in glycolysis mediated apoptosis of benzyl isothiocyanate in hepatocellular carcinoma
Source: Sci Rep. 2026 Feb 2;16:7000. doi: 10.1038/s41598-026-38416-8 (PMC12921019; doi:10.1038/s41598-026-38416-8)
Supplement: Supplementary file 1 — Supplementary Information. [file 41598_2026_38416_MOESM1_ESM.pdf]

Figure 2A

A

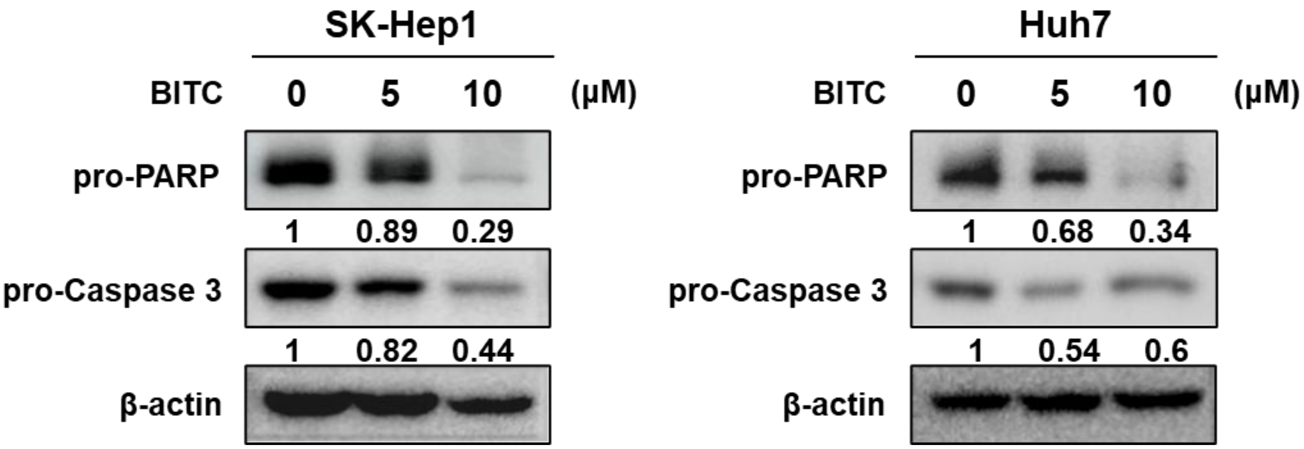
SK-Hep1
BITC 0 5 10 (μM)
pro-PARP
1 0.89 0.29
pro-Caspase 3
1 0.82 0.44
β-actin
Huh7
BITC 0 5 10 (μM)
pro-PARP
1 0.68 0.34
pro-Caspase 3
1 0.54 0.6
β-actin

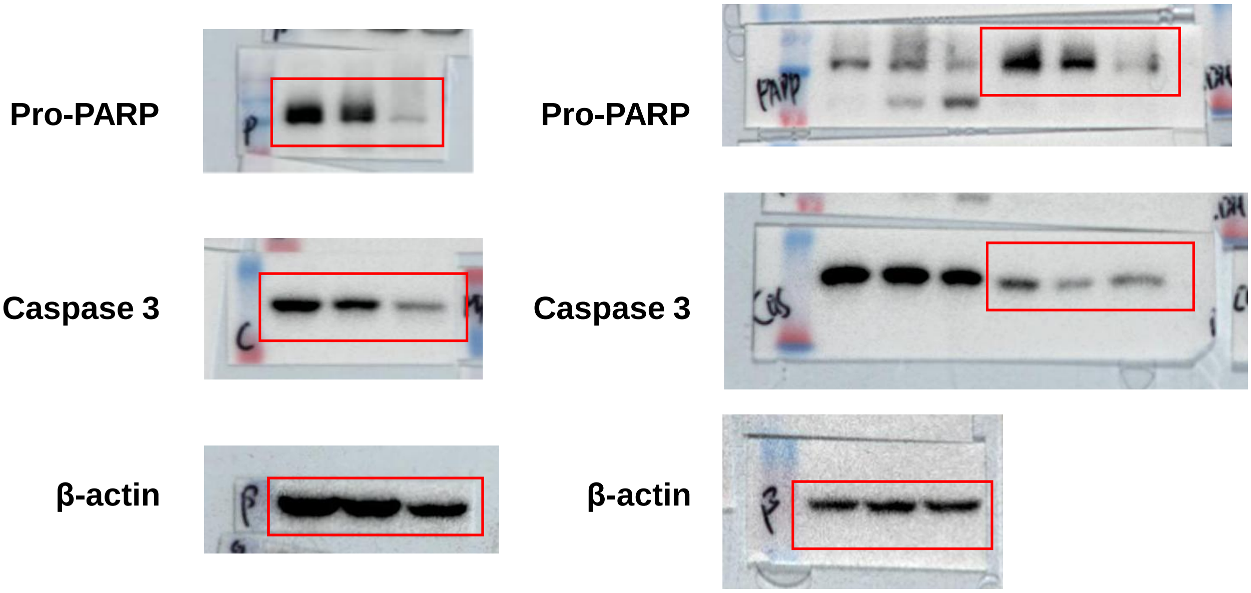
Pro-PARP
Caspase 3
β-actin
Pro-PARP
Caspase 3
β-actin

Figure 3B

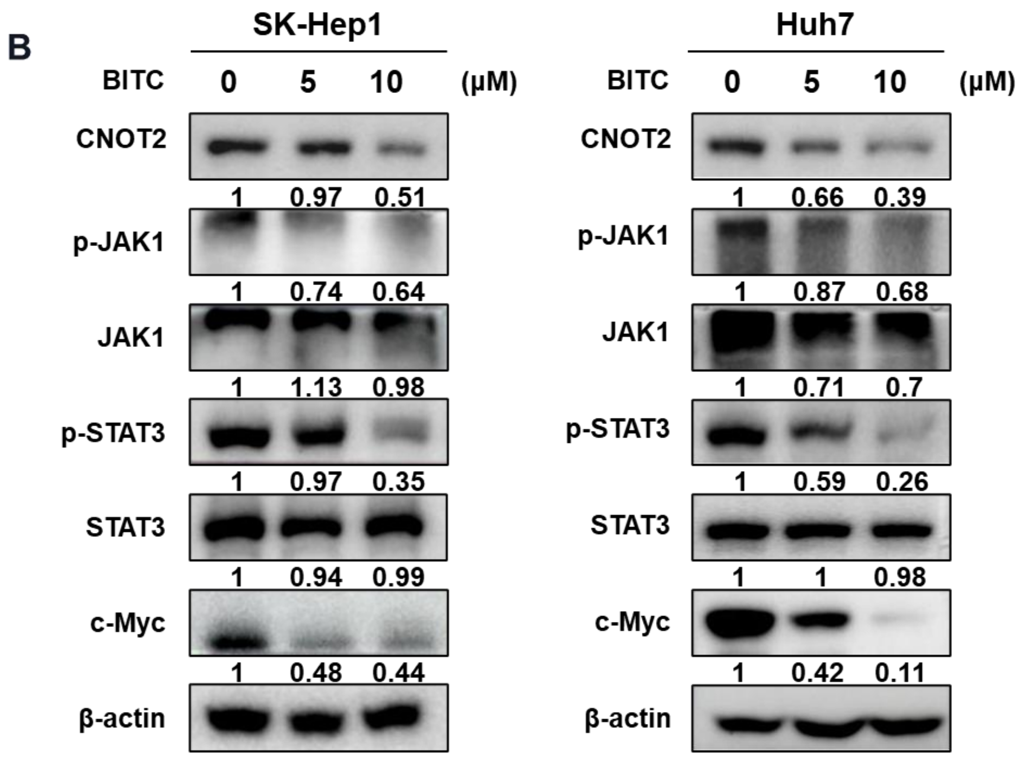

B
SK-Hep1
Huh7
BITC
0
5
10
(μM)
CNOT2
1 0.97 0.51
1 0.66 0.39
p-JAK1
1 0.74 0.64
1 0.87 0.68
JAK1
1 1.13 0.98
1 0.71 0.7
p-STAT3
1 0.97 0.35
1 0.59 0.26
STAT3
1 0.94 0.99
1 1 0.98
c-Myc
1 0.48 0.44
1 0.42 0.11
β-actin

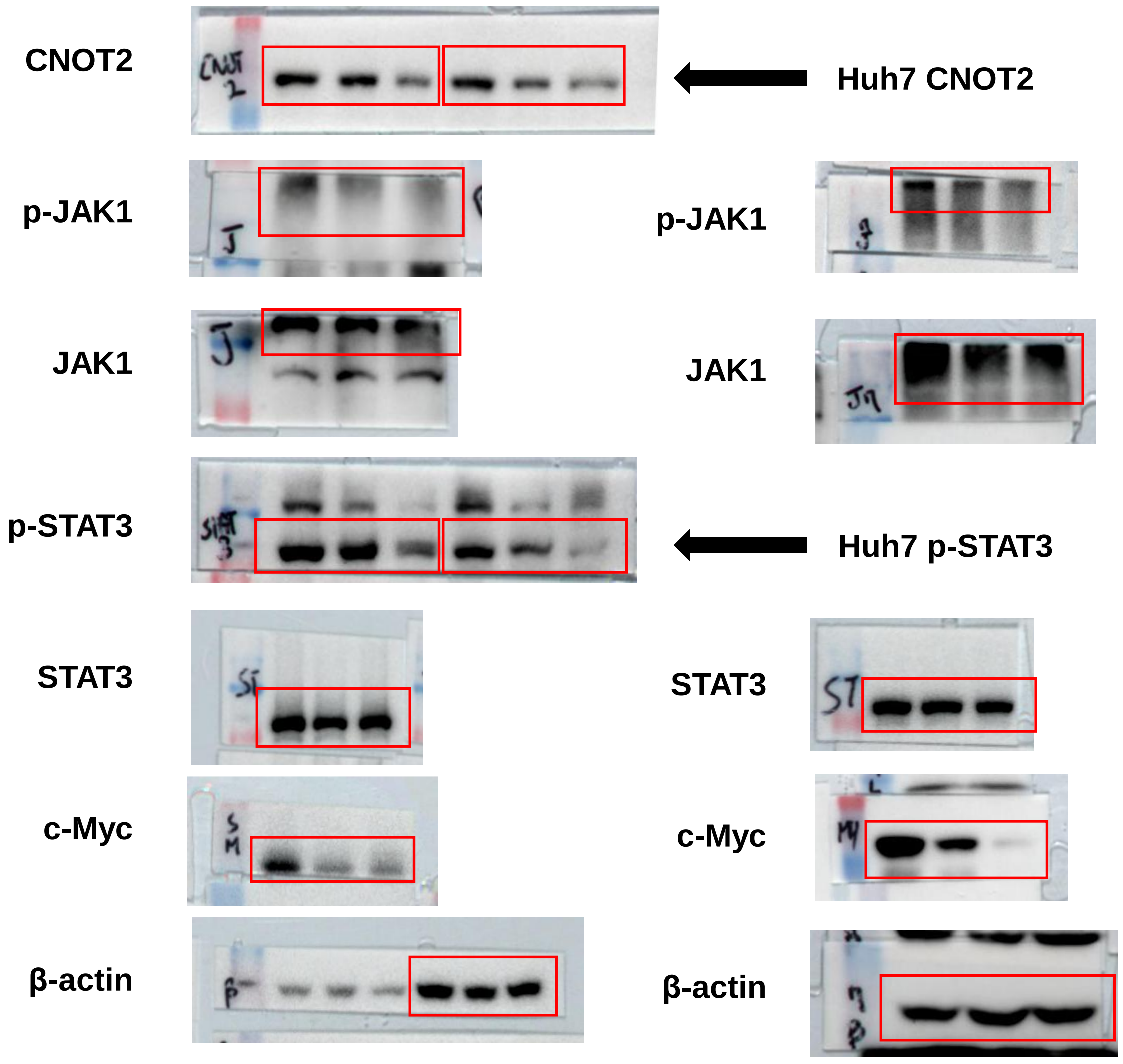

CNOT2
Huh7 CNOT2
p-JAK1
JAK1
p-STAT3
Huh7 p-STAT3
STAT3
c-Myc
β-actin

Figure 3C

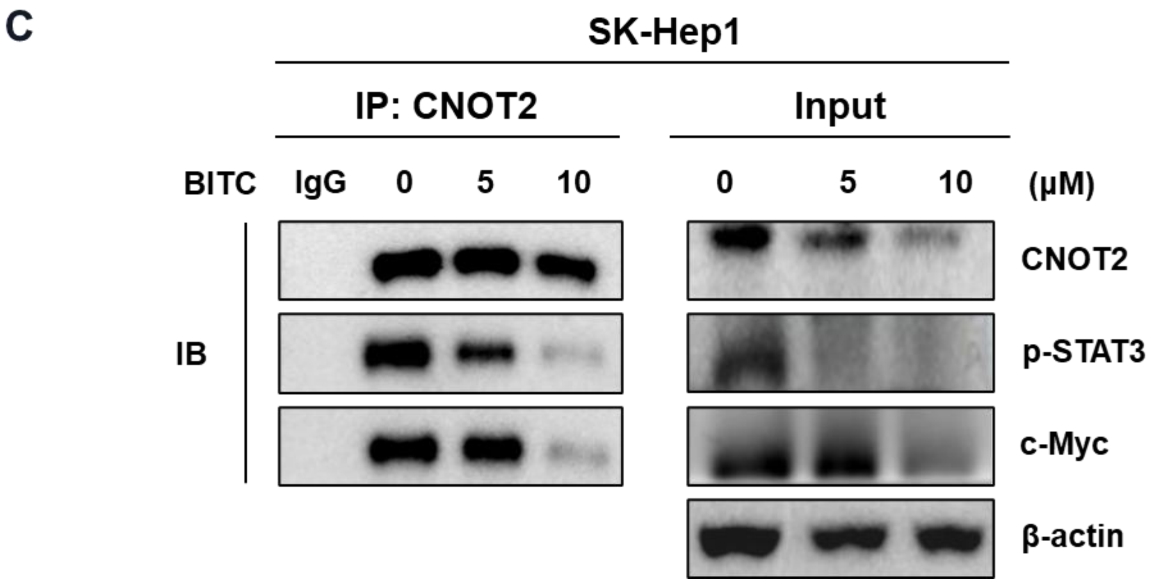
C
SK-Hep1
IP: CNOT2
Input
BITC
IgG
0
5
10
0
5
10
(μM)
IB
CNOT2
p-STAT3
c-Myc
β-actin

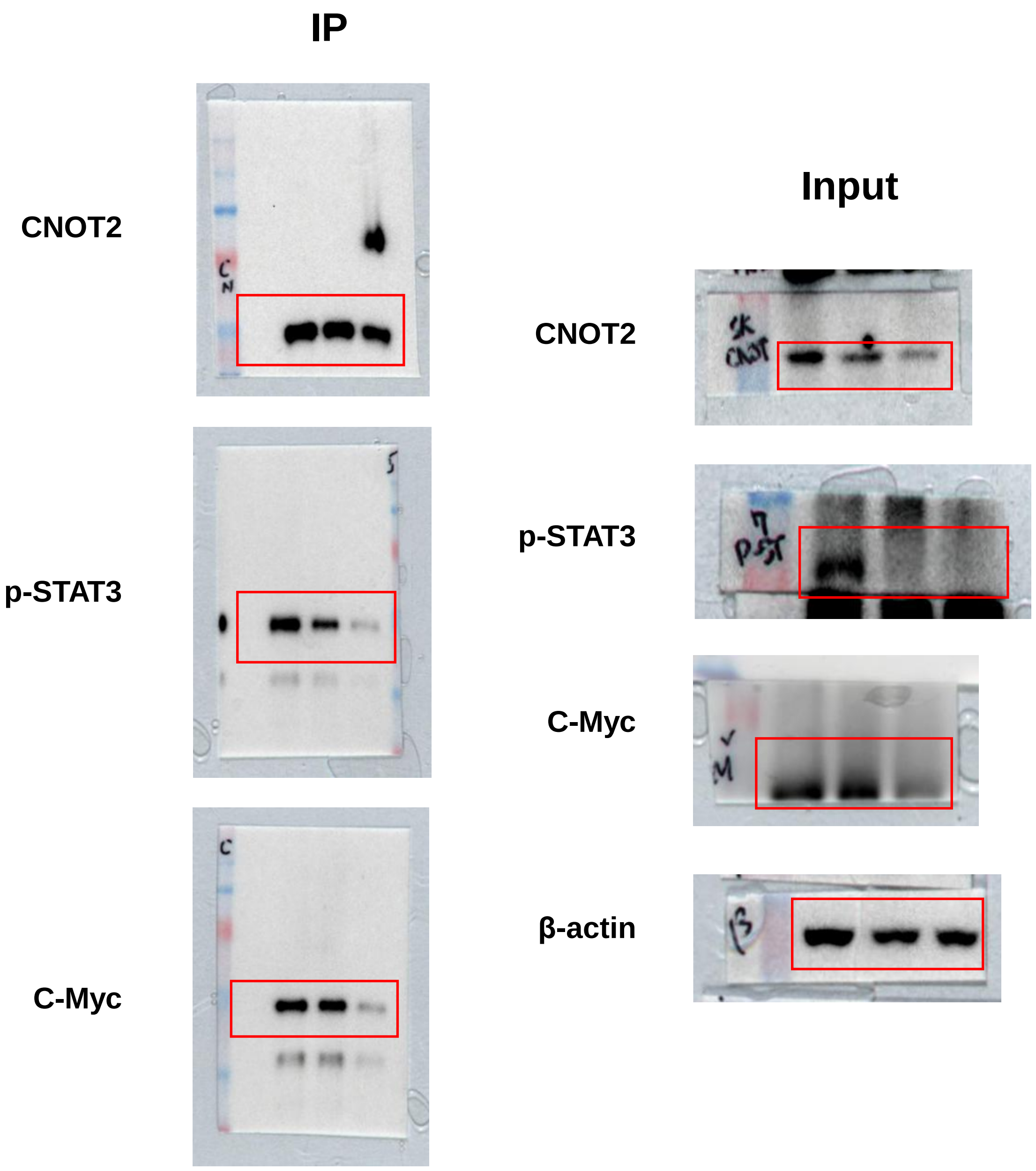
IP
CNOT2
p-STAT3
C-Myc
Input
CNOT2
p-STAT3
C-Myc
β-actin

Figure 4A

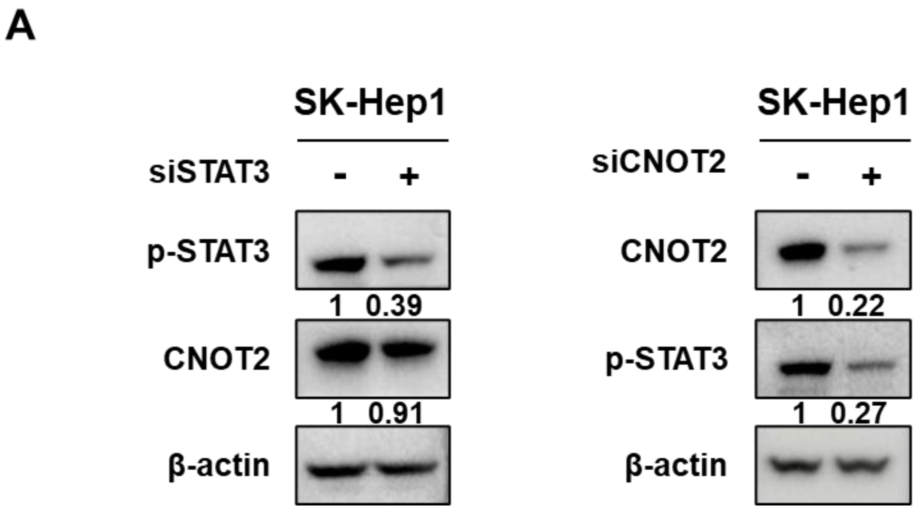
A
SK-Hep1
siSTAT3
-
+
p-STAT3
1 0.39
CNOT2
1 0.91
β-actin
SK-Hep1
siCNOT2
-
+
CNOT2
1 0.22
p-STAT3
1 0.27
β-actin

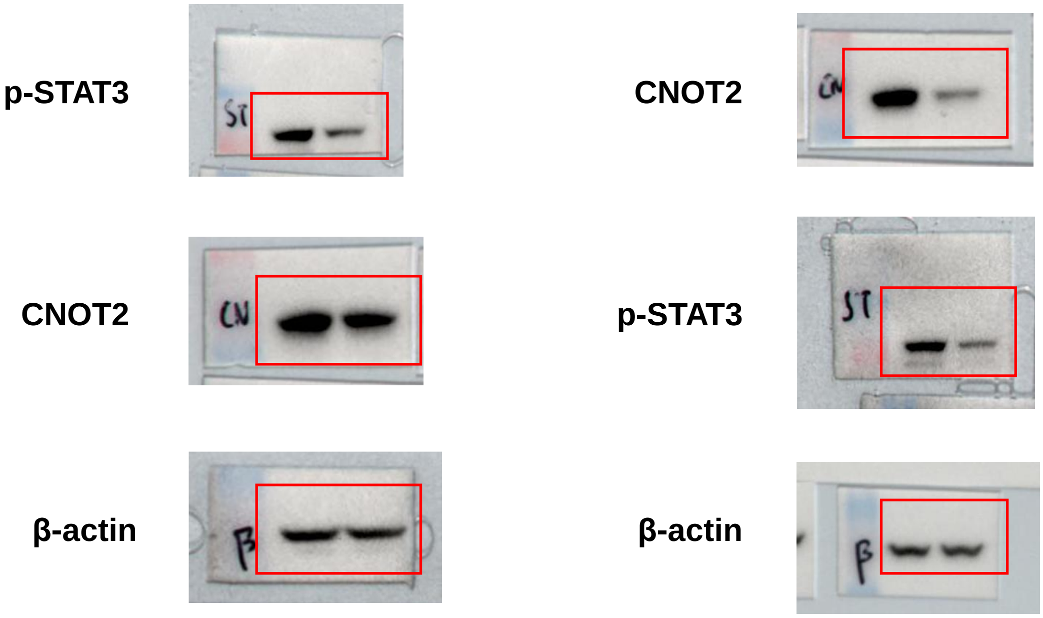
p-STAT3
CNOT2
β-actin
CNOT2
p-STAT3
β-actin

Figure 4B, C

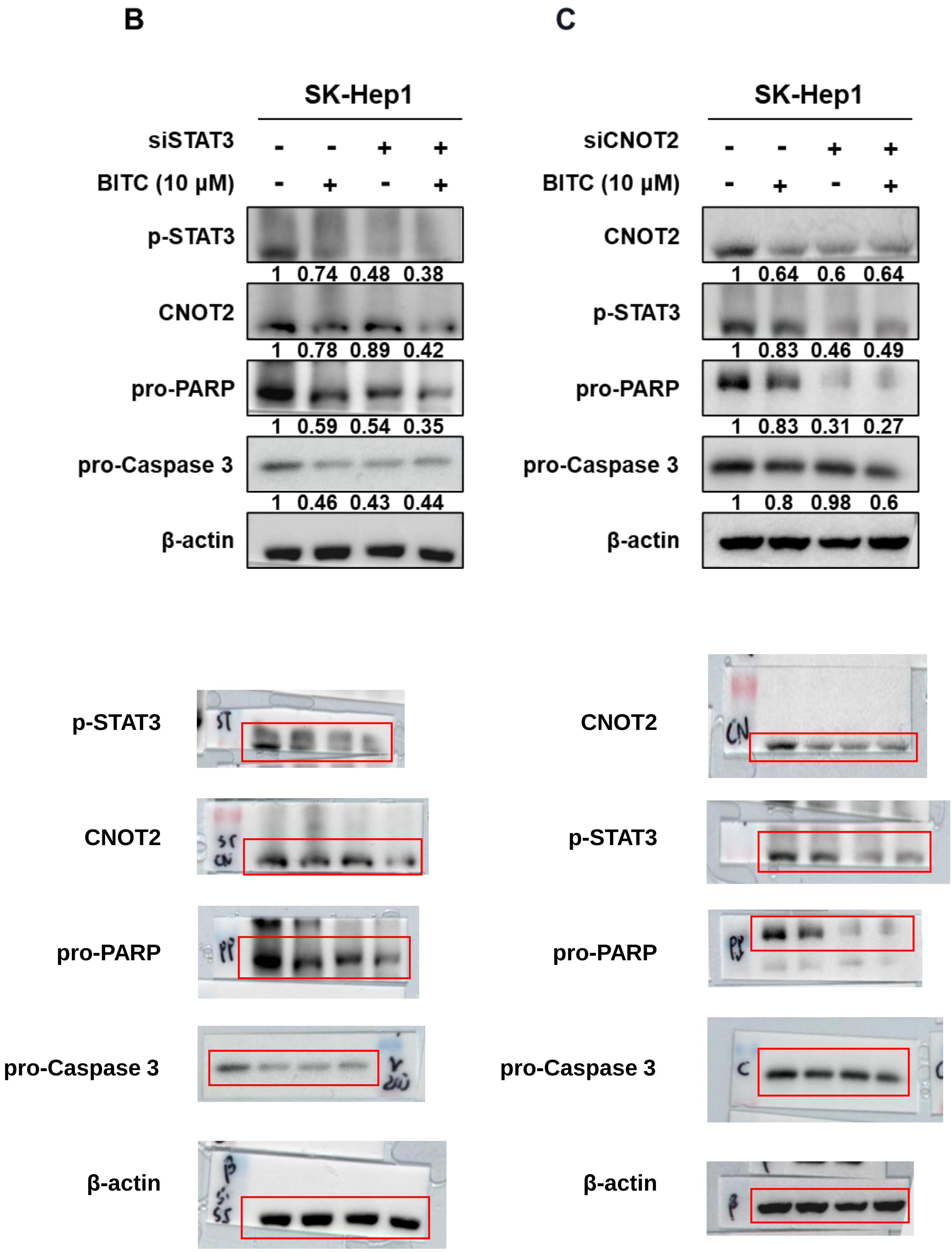
B
C
SK-Hep1
siSTAT3 - - + +
BITC (10 μM) - + - +
p-STAT3
1 0.74 0.48 0.38
CNOT2
1 0.78 0.89 0.42
pro-PARP
1 0.59 0.54 0.35
pro-Caspase 3
1 0.46 0.43 0.44
β-actin
SK-Hep1
siCNOT2 - - + +
BITC (10 μM) - + - +
CNOT2
1 0.64 0.6 0.64
p-STAT3
1 0.83 0.46 0.49
pro-PARP
1 0.83 0.31 0.27
pro-Caspase 3
1 0.8 0.98 0.6
β-actin
p-STAT3
CNOT2
pro-PARP
pro-Caspase 3
β-actin
CNOT2
p-STAT3
pro-PARP
pro-Caspase 3
β-actin

Figure 5A

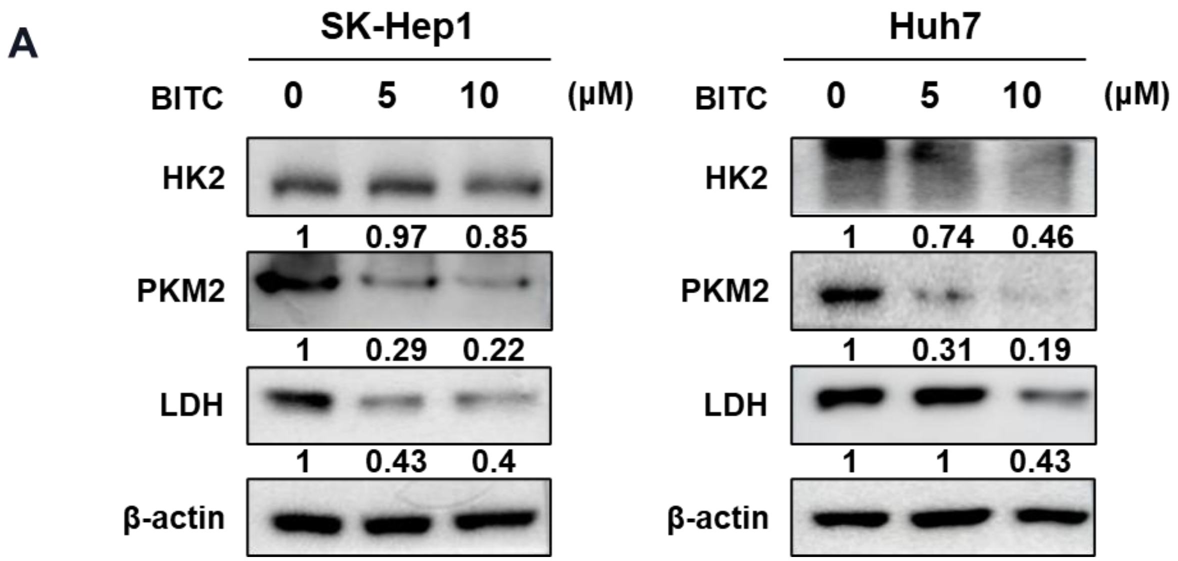
A
SK-Hep1
Huh7
BITC
0
5
10
(μM)
HK2
1 0.97 0.85
1 0.74 0.46
PKM2
1 0.29 0.22
1 0.31 0.19
LDH
1 0.43 0.4
1 1 0.43
β-actin

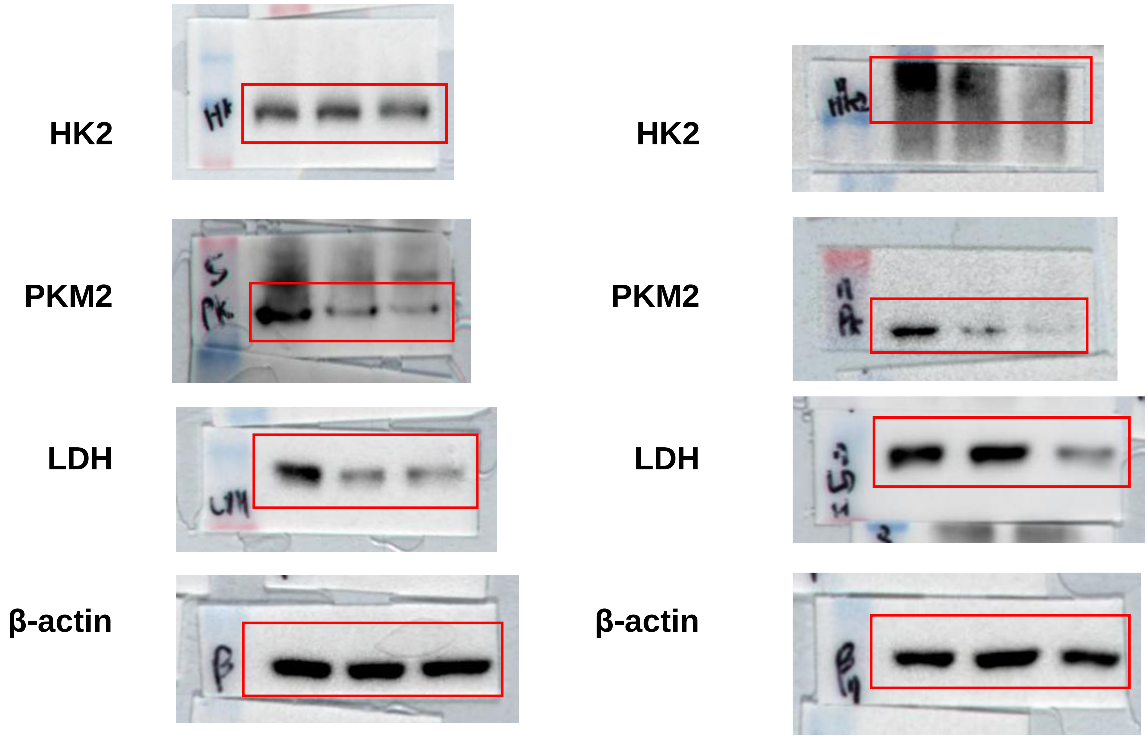
HK2
PKM2
LDH
β-actin

Figure 5D

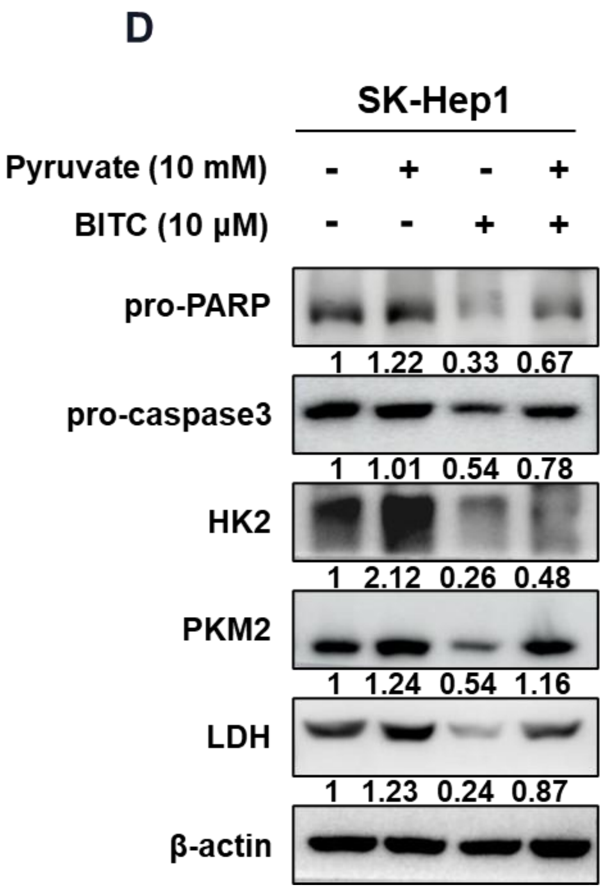

D
SK-Hep1
Pyruvate (10 mM) - + - +
BITC (10 μM) - - + +
pro-PARP
1 1.22 0.33 0.67
pro-caspase3
1 1.01 0.54 0.78
HK2
1 2.12 0.26 0.48
PKM2
1 1.24 0.54 1.16
LDH
1 1.23 0.24 0.87
β-actin

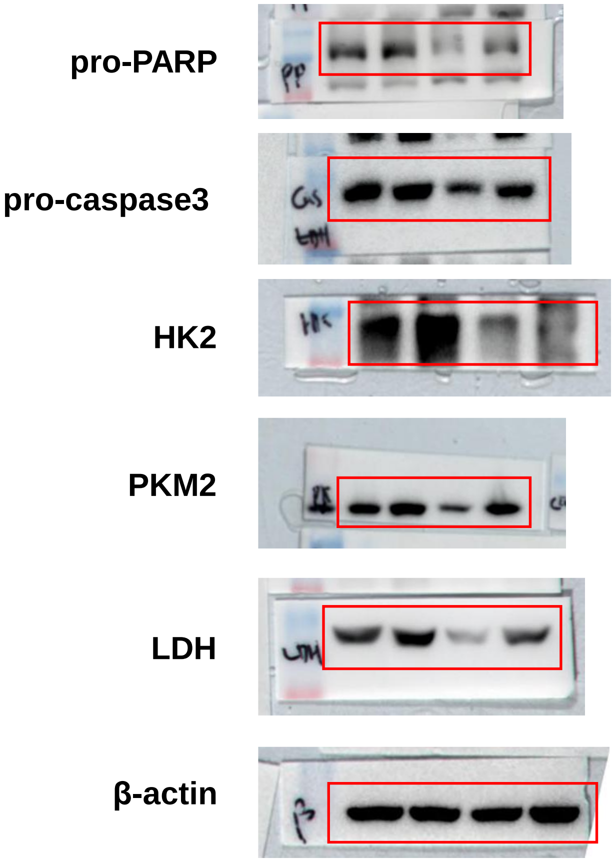

pro-PARP
pro-caspase3
HK2
PKM2
LDH
β-actin

Figure 5D

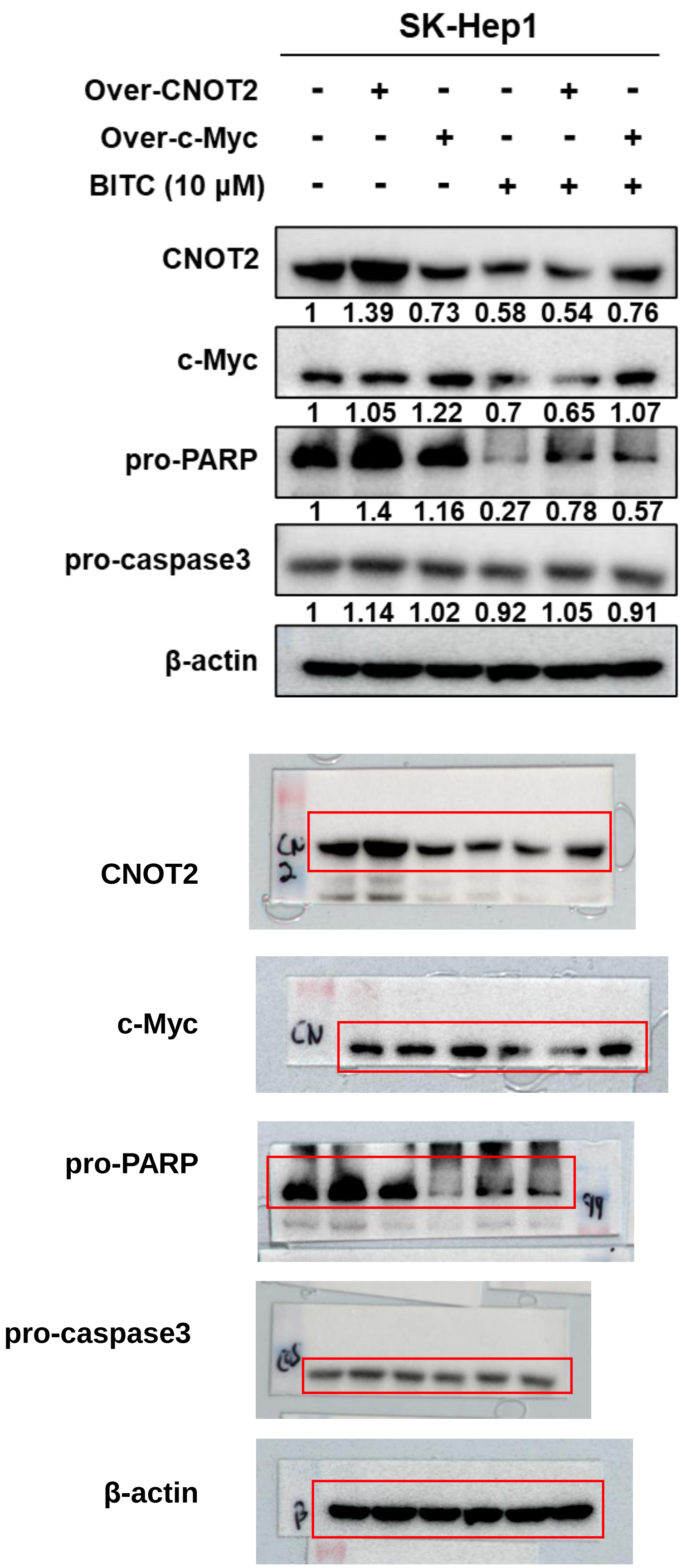
SK-Hep1
Over-CNOT2 - + - - + -
Over-c-Myc - - + - - +
BITC (10 μM) - - - + + +
CNOT2
1 1.39 0.73 0.58 0.54 0.76
c-Myc
1 1.05 1.22 0.7 0.65 1.07
pro-PARP
1 1.4 1.16 0.27 0.78 0.57
pro-caspase3
1 1.14 1.02 0.92 1.05 0.91
β-actin
CNOT2
c-Myc
pro-PARP
pro-caspase3
β-actin

Figure 5E

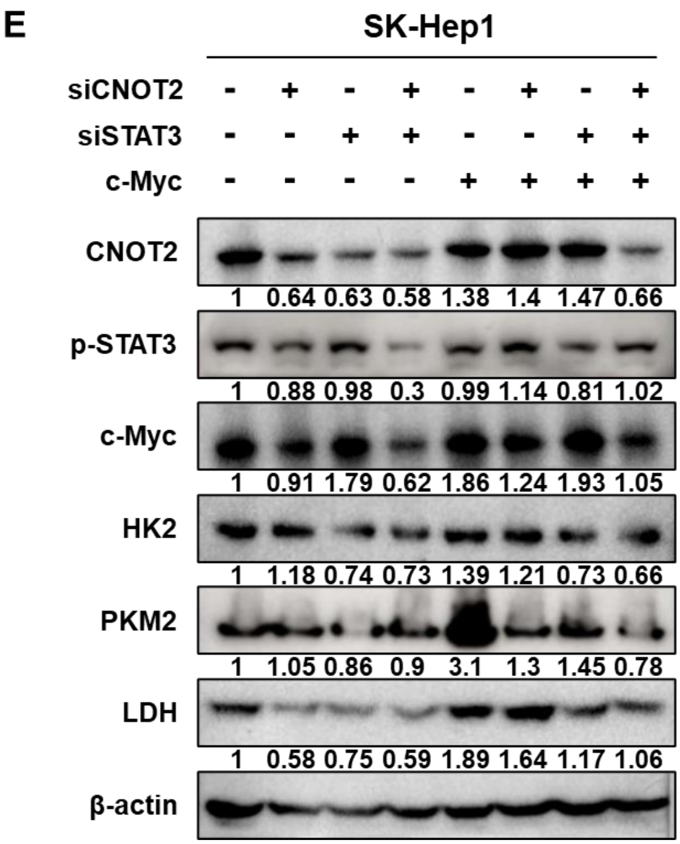

E
SK-Hep1
siCNOT2 - + - + - + - +
siSTAT3 - - + + - - + +
c-Myc - - - - + + + +
CNOT2
1 0.64 0.63 0.58 1.38 1.4 1.47 0.66
p-STAT3
1 0.88 0.98 0.3 0.99 1.14 0.81 1.02
c-Myc
1 0.91 1.79 0.62 1.86 1.24 1.93 1.05
HK2
1 1.18 0.74 0.73 1.39 1.21 0.73 0.66
PKM2
1 1.05 0.86 0.9 3.1 1.3 1.45 0.78
LDH
1 0.58 0.75 0.59 1.89 1.64 1.17 1.06
β-actin

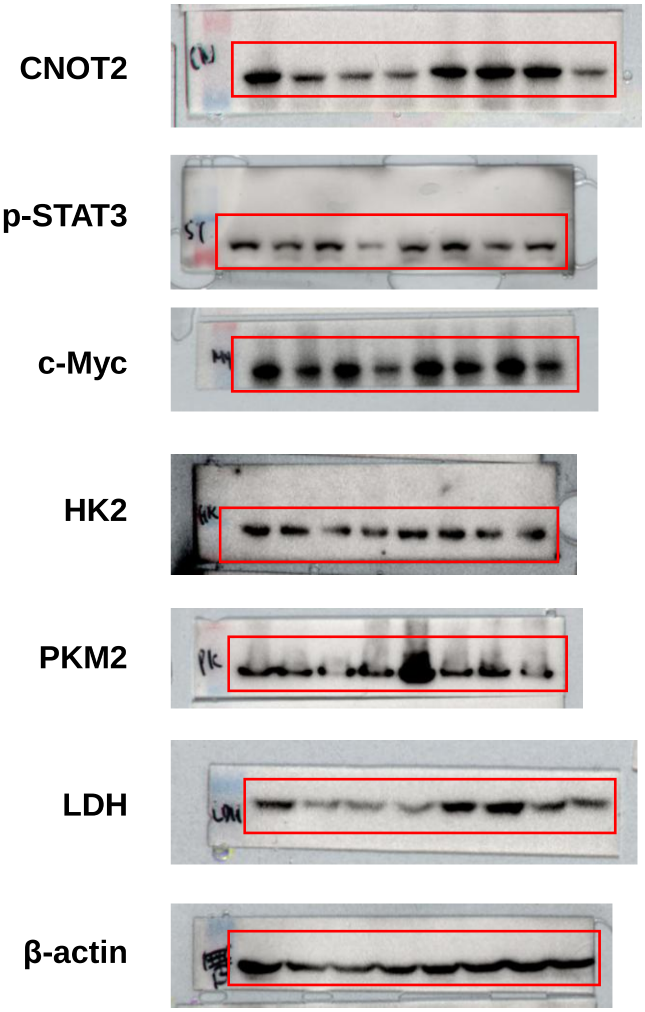

CNOT2
p-STAT3
c-Myc
HK2
PKM2
LDH
β-actin
